# Ligand-Dependent and -Independent Functions of Activation Function 1 of Progesterone Receptor in Genome-Wide Gene Regulation and in Cell Proliferation and Apoptosis of Breast Cancer Cells

**DOI:** 10.3390/ijms27062916

**Published:** 2026-03-23

**Authors:** Pheck Khee Lau, Bernett Lee Teck Kwong, Shi Hao Lee, Chew Leng Lim, Qian Yee Woo, Amanda Rui En Woo, Jace Koh, Valerie C. L. Lin

**Affiliations:** 1 School of Biological Sciences, Nanyang Technological University, Singapore 637511, Singapore; pheckkhee.lau@ntu.edu.sg (P.K.L.); leeshifu@gmail.com (S.H.L.); clim050@e.ntu.edu.sg (C.L.L.); qianyee.woo@ntu.edu.sg (Q.Y.W.); wooruien@gmail.com (A.R.E.W.); jace0007@e.ntu.edu.sg (J.K.); 2 Lee Kong Chian School of Medicine, Nanyang Technological University, Singapore 637511, Singapore; bernett.lee@ntu.edu.sg

**Keywords:** progesterone receptor, activation function 1, transcriptional activity, geneome-wide gene regulation, breast cancer

## Abstract

Progesterone receptor (PR) regulates gene expression through recruiting coregulators and general transcription factors by activation functions AF1 and AF2. AF1 localizes to the non-conserved and disordered N-terminal domain and is believed to facilitate tissue- and gene-specific activity. Our previous proteomic analysis identified three key residues (K464, K481 and R492) in AF1 that are monomethylated. Methylation mimic mutations KKR → FFF created hypoactive PR, whereas the KKR → QQQ mutation generated hyperactive PR in gene reporter assays. The current study used these mutants to determine the roles of AF1 in PR regulation of cellular activities and global gene regulation in breast cancer cells MCF-7. AF1-FFF mutation attenuated PR regulation of cell proliferation and apoptosis in response to progestin, whereas AF1-QQQ mutation enhanced these effects. AF1-FFF mutation attenuated gene regulation by progestin in ~60% of PR target genes, including genes involved in cell proliferation, hypoxia and TNFα signaling. However, the AF1-FFF mutation had little effect on ligand-independent gene regulation, suggesting distinct mechanisms of gene regulation by liganded and unliganded PR. Intriguingly, impaired activity of methylation mimic mutant PRB-FFF is associated with greater chromatin binding in ChIP-Seq analysis, corresponding to a stronger association between PRB-FFF and Steroid Receptor Coactivator-1 (SRC-1), a member of the p160 family of nuclear receptor coactivators, as was previously reported. In conclusion, PR AF1 is important for the core activities of liganded PR in regulating ~half of target genes and cell proliferation. AF1 monomethylation may modulate PR-chromatin interactions through stronger association with coregulators, thereby decelerating chromatin binding kinetics. This is supported by PRODIGY’s prediction of higher binding affinities of monomethylated AF1 and methylation mimic mutant with SRC-1.

## 1. Introduction

Progesterone is critical for embryo implantation and maintenance of pregnancy through coordinated antagonistic and synergistic effects with estrogen on the endometrium [1]. This involves inhibition of estrogen-induced luminal epithelial cell proliferation in the preimplantation window and stimulation of proliferation and differentiation of uterine stromal cells during embryo implantation. The antiproliferative effect of progesterone on luminal cells plays an important role in preventing endometriosis and endometrial cancer [2]. Progesterone is also involved in mammary stem cell expansion in the adult and is critical for mammary ductal branching and alveologenesis [3,4]. However, its role in breast cancer has been controversial. Hormone replacement therapy (HRT) trials initially reported increased risk of breast cancer for women taking estrogen plus progestin compared to those taking estrogen alone [5,6], but follow-up reports and subsequent critical reviews indicated little association between the use of progestin in HRT and breast cancer [7,8,9,10]. Consequently, there has been a revival of interest in progesterone receptor (PR) targeting therapies for breast cancer. Further understanding of the molecular mechanism of progesterone action is important for informed decision-making on PR targeted therapy.

PR exists in two main isoforms, PRA and PRB, which are transcribed from different promoters, resulting in an additional 164 amino acids in PRB at its N terminus. PR is composed of a modular domain structure with an N-terminal domain (NTD), DNA-binding domain (DBD), hinge region (H), and ligand-binding domain (LBD) [11]. Like all NRs, the transcriptional activity of PR is primarily mediated by Activation Function AF1 and AF2. AF1 is located within the intrinsically disordered NTD and was found initially to be a weak ligand-independent activation domain [12], while AF2 was located in the highly conserved and structured LBD [13]. PRB has an additional AF3 in its N terminus. It is known that AF1 and AF2 interact and synergize to mediate ligand-induced transcriptional activity through recruiting transcription coregulators, such as the p160 family of coactivators SRC-1, SRC-2 and SRC-3, histone acetylase p300/CREB-binding protein (CBP), and corepressors such as NCoR1 and SMRT, which serve to recruit chromatin remodelers and the general transcriptional machinery [14,15]. Structural study of PRB complex with coactivators SRC-2 and p300 protein showed that PRB homodimer engages one SRC-2 through AF1 and binds to one p300 through AF2 and its unique AF3 [16]. It indicates that PR-B adopts a distinct coactivator assembly from other steroid hormone receptors, such as estrogen receptor alpha (ERα) and androgen receptor. Dynamics of interaction between AF1/AF2 and coregulators can conceivably influence gene regulation.

The non-conserved and intrinsically disordered nature of AF1 renders it structural plasticity, allowing malleable interaction and partner protein-induced folding. For example, binding of TATA-binding protein (TBP) to glucocorticoid receptor AF1 induces AF1 folding and facilitates its interaction with SRC-1 [17]. This structural plasticity and flexible interactions are said to be the basis for its cell and gene-specific activities. However, the extent of AF1’s involvement in cell- and gene-specific activity is still poorly understood. Unlike the highly conserved AF2, the primary sequence of AF1 is highly diverse among NRs, making it challenging to identify critical functional residues. Proteomic profiling of post-translational modifications of PR from T47D cells revealed three monomethylation sites, K464, K481, and R492 on AF1. Mutagenesis analysis showed that K464, K481 and R492 function cooperatively [18,19]. Methylation mimic mutation KKR → FFF largely abolished PR activity, while the KKR → QQQ mutation renders PR hyperactive in reporter gene assays. Mice with the KKR-FFF mutation are infertile due to implantation and decidualization defects [20]. These mice also exhibit defective mammary alveologenesis associated with low RANKL expression [21]. These in vivo data suggest that the activity of PR AF1 is important for cell growth and differentiation, although detailed mechanisms are still lacking.

Targeting the AF1 of steroid hormone receptors is a promising strategy for cancer treatment. AF1 of androgen receptors has been extensively studied for targeting prostate cancer with small molecule inhibitors [22,23,24]. A deeper insight into the regulatory functions of PR AF1 in breast cancer is important for advancing PR-targeted therapies. The current study evaluated the role of PR AF1 in the regulation of breast cancer cell growth and death, and determined its extent of involvement in PR regulation of genome-wide gene expression and PR-chromatin interaction with or without progestin. The study also elucidated the role of AF1 in the functional interplay on gene regulation between PR and ERα.

## 2. Results

### 2.1. Stable Expression of PR and AF1 Mutants Through Lentiviral Transduction

**Figure 1:**
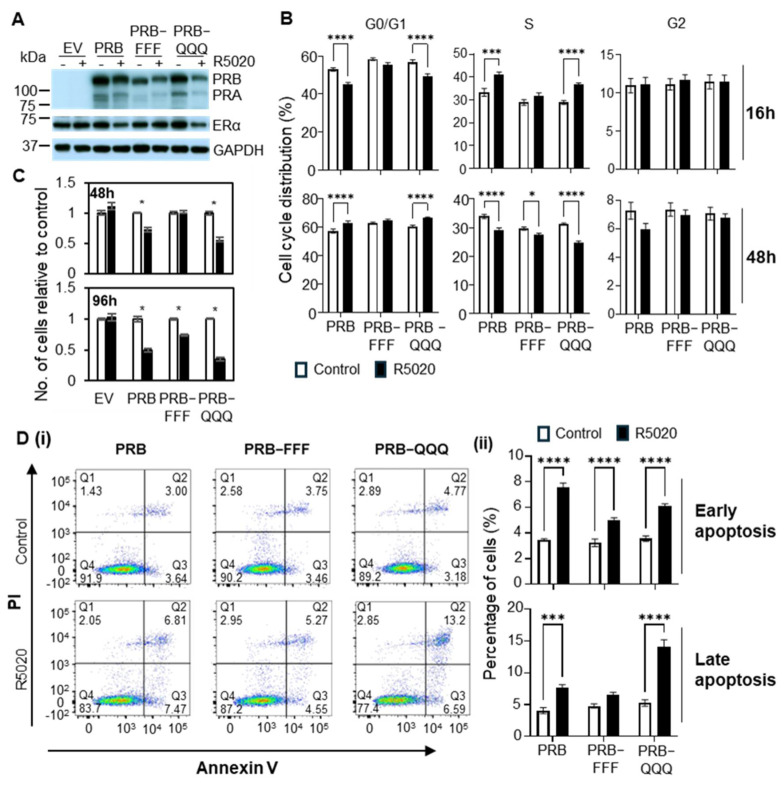
**AF1 is required for PR to regulate cell growth and apoptosis.** (**A**) Western blotting analysis of PR and Erα protein levels in MCF-7 cells transduced with PRB and AF1 mutant cDNA. GAPDH was probed as a loading control. All Western blot experiments were performed in biological duplicates. (**B**) Effect of R5020 on cell cycle progression measured by flow cytometry. Percentages of cells in G0/G1, S and G2/M phase after 16 h and 48 h of R5020 treatment from four independent experiments (*n* = 12) are presented. (**C**) Cell number after 48 h and 96 h of R5020 treatment in PRB, PRB-FFF and PRB-QQQ cells. Empty vector transduced cells (EV) served as controls. The data are presented as mean ± SEM (*n* = 6 from two independent experiments) and are expressed relative to the vehicle-treated cells, which is set to the value of 1. (**D**) AF1-FFF mutations attenuated R5020-induced apoptosis. (**D(i)**) Gating of early (Q3) and late (Q2) apoptotic cells. (**D(ii)**) Percentage of early (Q3) and late (Q2) apoptotic cells after 96 h R5020 treatment in PRB, PRB-FFF and PRB-QQQ cells is presented (*n* = 6 from two independent experiments). Asterisks indicate statistical significance between control and R5020-treated samples (* *p* < 0.05, *** *p* < 0.001, **** *p* < 0.0001).

We reported that PRB-FFF and PRB-QQQ exhibited hypoactivity and hyperactivity in the reporter gene assay, respectively [19]. To evaluate the effects of the mutations on specific molecular and cellular activity of AF1 in breast cancer cells, stable expression of wild-type PRB, PRB-FFF, and PRB-QQQ was established through lentiviral transduction in MCF-7 cells, and all cells stably transduced with PR or mutant cDNA were selected in hygromycin-containing medium and propagated into cell lines. Cells transduced with the empty viral vector (EV) were established as transfection controls. Both PRB-FFF and PRB-QQQ mutants exhibit normal nuclear localization characteristics (Supplementary Figure S1). The immunoblot in Figure 1A shows that the PRB is the main isoform and PRA is expressed at a much lower level (~15%) from the second ATG of the PRB cDNA. This is largely agreeable with the relatively lower PRA protein level in the commonly studied breast cancer cell line T47D [25] and estrogen-treated MCF-7 cells [26], in which the PRA level is ~25% of PRB. Protein levels of PRB-FFF were slightly lower than those of PRB and PRB-QQQ. This is possibly due to a greater proportion of PRB-FFF associated with chromatin, as will be shown in ChIP-Seq data later. R5020 downregulated the level of ERα in PRB cells, and this effect remained in PRB-QQQ cells but lost in PRB-FFF cells. This downregulation of ERα by R5020-activated PR has been reported in our previous study and is found to play a key role in the anti-estrogenic effect of R5020 in MCF-7 cells mediated through diminished chromatin-associated ERα and FOXA1 [27]. Notably, endogenous PR was not observed in the EV cells because its expression is estrogen-dependent in MCF-7 cells and the phenol red free mediate with charcoal-stripped serum lacked estrogenic activity to induce PR.

### 2.2. AF1 Is Important for PR to Regulate Cell Growth and Apoptosis

These MCF-7-derived cell lines with PRB or PRB AF1 mutants were then used to evaluate the molecular and cellular activities of AF1. R5020 (promegestone) is a synthetic progestin and highly selective PR agonist. It is commonly used to study PR activity in response to a ligand. R5020 is known to exert a biphasic effect on the cell cycle progression of breast cancer cells T47D and MCF-7 with PR overexpression, in which R5020 accelerates G0/G1 to S phase progression initially, followed by G0/G1 arrest after 24–36 h treatment [27,28,29]. Accordingly, R5020 reduced G0/G1 phase cells and increased S phase cells after 16 h treatment of PRB cells (Figure 1B), indicating that R5020 promoted cell cycle acceleration. After 48 h treatment, R5020 increased the fraction of G0/G1 cells and reduced the S phase fraction, indicating cell cycle arrest at the G0/G1 phase. R5020 showed a similar biphasic effect on PRB-QQQ cells. However, R5020 was unable to accelerate G0/G1 progression to S phase after 16 h in PRB-FFF cells, and its effect on G0/G1 phase arrest after 48 h was impaired in PRB-FFF cells compared to PRB cells.

The effect of R5020 on cell proliferation was also determined by cell counting. R5020 reduced the cell number of PRB cells by 25% and 50% after 48 h and 96 h treatment, respectively (Figure 1C). PRB-QQQ cells showed hyperactivity by reducing cell number by 40% and 65%. In contrast, R5020 treatment for 48 h did not reduce cell number in PRB-FFF cells, and it reduced cell number by only 25% after 96 h treatment, confirming the importance of AF1 in growth regulation in response to progestin.

AF1 mutations also altered the effect of progestin on cell morphology. R5020-induced growth inhibition was associated with cell spreading in PRB and PRB-QQQ cells after 96 h treatment (Supplementary Figure S2). In contrast, R5020-induced cell spreading in PRB-FFF cells was visibly less than that of PRB and PRB-QQQ cells. Thus, AF1 is also involved in progestin-induced signaling to cell adhesion.

Apoptosis is a highly regulated form of programmed cell death and a stage-dependent process [30]. Early apoptosis involves reversible cell shrinkage and Phosphatidylserine (PS) flipping to the outer membrane (Annexin V positive but PI negative). Late apoptosis exhibits irreversible DNA fragmentation and loss of membrane integrity (Annexin V positive and PI positive). Early apoptosis usually proceeds to late apoptosis if an apoptotic stimulus persists. We reported that treatment with R5020 induces marked apoptosis in MCF-7 cells stably overexpressing PRB [27]. Here, we tested if AF1 is involved in R5020-induced apoptosis. After 96 h treatment, R5020 also induced early apoptosis significantly in PRB, PRB-FFF and PRB-QQQ cells, but it did not significantly induce late apoptosis in PRB-FFF cells (Figure 1D). This reflects a slower onset of apoptosis in PRB-FFF cells. Together, the methylation mimic mutant PRB-FFF exhibited loss of activity in regulating cell proliferation and apoptosis in response to progestin, indicating critical roles of AF1 in regulating these cellular activities.

### 2.3. AF1 Activity Is Gene-Specific and Important for Regulation of Cell Proliferation and Apoptosis

#### 2.3.1. Hypoactivity of PRB-FFF and Hyperactivity of PRB-QQQ in Subsets of PR Target Genes

To investigate the genome-wide activity of AF1, bulk RNA-Seq analysis was conducted after 6 h of R5020 treatment. The relatively short 6 h treatment is chosen to capture the regulation of primary target genes of PR. Data quality and reproducibility were validated using Principal Component Analysis (PCA) and Pearson correlation across all biological replicates. PCA demonstrated that samples clustered primarily by treatment and cell line, with biological replicates grouping tightly together, while EV showed random clustering due to a lack of response to R5020 (Supplementary Figure S3A). Pearson correlation analysis further confirmed high reproducibility, with coefficients (R^2^) exceeding 0.99 for every replicate pair across all cell lines.

**Figure 2:**
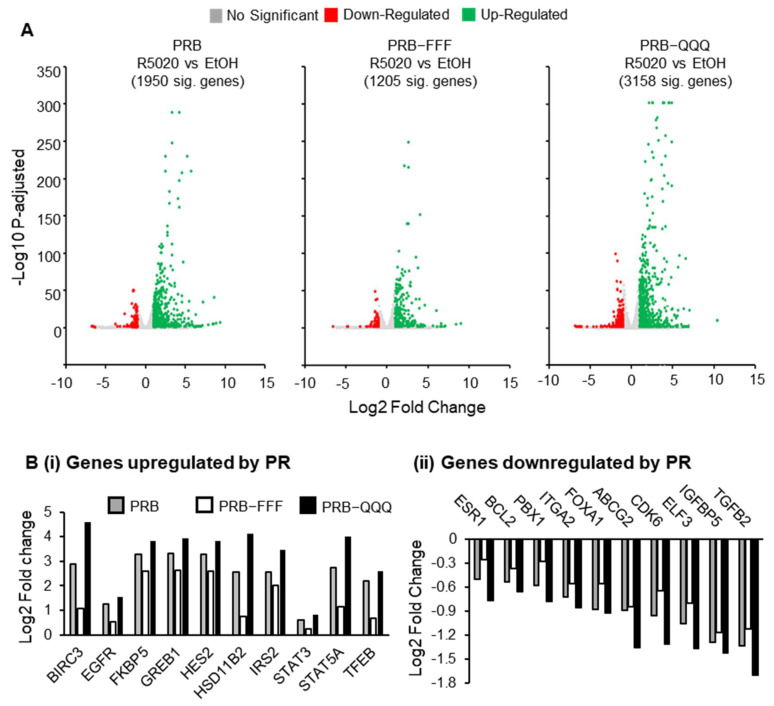
**PRB-FFF cells exhibit hypoactivity while PRB-QQQ cells exhibit hyperactivity in gene regulation in response to R5020.** (**A**) Volcano plots show significantly regulated genes (padj < 0.05) are fewer in number and magnitude in PRB-FFF cells and more in PRB-QQQ cells than in PRB cells. (**B**) Attenuated and enhanced regulation of well-characterized PR upregulated (**i**) and downregulated (**ii**) genes in PRB-FFF and PRB-QQQ cells, respectively. The fold change values represent the ratio of R5020-treated to vehicle control (EtOH).

DESeq2 analysis revealed 1950, 1205, and 3158 significantly regulated genes (padj < 0.05) by PRB, PRB-FFF, and PRB-QQQ, respectively. Volcano plots show that the R5020-induced fold changes and statistical significance in PRB-FFF cells are generally lower, while those in PRB-QQQ cells are generally higher than those in PRB cells (Figure 2A).

Of the 1950 PRB-regulated genes (padj < 0.05), 843 (43%) were not regulated by PRB-FFF with padj > 0.1, while another 309 (16%) were hypo-regulated by PRB-FFF by more than 20%. Together, the AF1-FFF mutation substantially hindered AF1 activity on ~60% of PR target genes. In contrast, 1726 genes (89%) remained significantly regulated by the PRB-QQQ mutant with padj > 0.1. Among these genes, 300 (15%) exhibited hyper-regulation, and 70 (3.6%) exhibited hypo-regulation by more than 20% relative to PRB. These gene expression data are generally consistent with the hypo- and hyperactivity of PRB-FFF and PRB-QQQ in the PR report gene assay [19], although the effect of AF1-QQQ mutation was more gene selective. It is worth noting that the well-characterized PR target genes such as *FKBP5*, *HSD11B2*, *STAT5A*, *ESR1* and *FOXA1* were commonly hypo-regulated and hyper-regulated by PRB-FFF and PRB-QQQ, respectively (Figure 2B(i,ii)), indicating important roles of AF1 on these genes.

On the other hand, the expression of 541 genes out of 1950 R5020-regulated genes (~28%) was not affected by AF1-FFF mutation by more than 10% (Supplementary File S1). GSEA or GO analysis of gene expressions not affected by AF1-FFF mutation did not yield significant enrichment of pathways or functional categories, indicating these genes are involved in diverse molecular functions. For example, functions of the top 10 progesterone-regulated genes in this list include cell adhesion and calcium signaling (*AHNAK*), regulation of morphogenesis (*GRHL2*), metabolism and bioenergetics (*ACSS1*, *DDIT4*, and *SLC5A6*), and protein ubiquitination (*NEDD4L*) (Supplementary Figure S3B). Taken together, AF1 activity is gene-specific, and the hypoactive AF1-FFF mutation attenuated regulation in about 60% PR target genes.

#### 2.3.2. AF1-FFF Mutation Cause Loss of Overrepresentation of PR Regulated Hallmarks, Including Hypoxia

**Figure 3:**
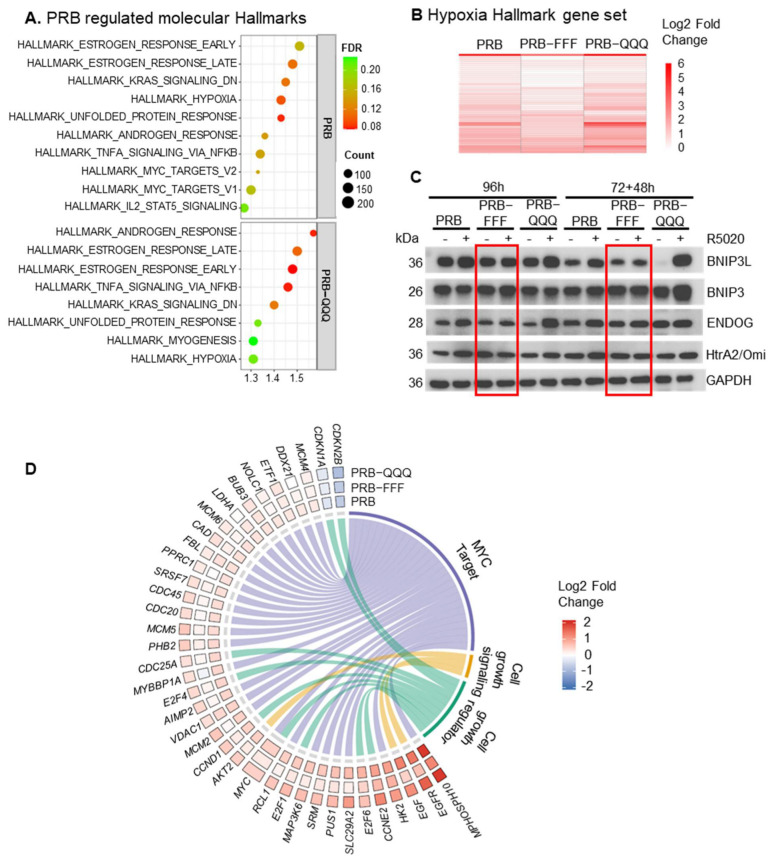
**Regulatory activity of AF1 is broadly important and specifically in regulation of hypoxia and cell proliferation.** (**A**) No GSEA hallmark gene set was significantly enriched by R5020 in PRB-FFF cells. The bubble plot shows hallmark gene sets with FDR < 0.25 and enrichment score > 1.2 enriched in PRB and PRB-QQQ cells. (**B**) Heatmap of R5020 induced log2fold change for genes associated with HALLMARK_HYPOXIA. More than 80% of PR target genes in hypoxia are hypo-regulated by PRB-FFF relative to PRB, suggesting loss of function. The fold change values represent the ratio of R5020-treated to vehicle control (EtOH) within each respective cell line. (**C**) PRB-FFF failed to induce BNIP3L and BNIP3, which are key players in hypoxia and apoptosis. Protein levels of BNIP3L, BNIP3, ENDOG and HtrA2/Omi were analysed by Western blotting analysis. R5020-induced increase of these proteins in PRB and PRB-QQQ cells, but not in PRB-FFF cells (in red box). GAPDH was probed as a loading control. All Western blot experiments were performed in biological duplicates. (**D**) Chord diagram showing attenuated regulation of cell cycler regulators in PRB-FFF cells compared with PRB and PRB-QQQ cells, consistent with delayed cell cycle progression.

Gene set enrichment analysis (GSEA) was conducted to see whether these AF1-regulated genes belong to specific functional categories or are in specific pathways. Positively enriched hallmark gene sets with FDR < 0.25 and normalized enrichment score > 1.2 were listed in Figure 3A. Common hallmark gene sets enriched in both PRB and PRB-QQQ cells include ESTROGEN_RESPONSE_EARLY, ESTROGEN_RESPONSE_LATE, KRAS_SIGANLLING_DN, TNFA SIGNALING VIA NFKB, HYPOXIA, and UNFOLDED_PROTEIN_RESPONSE. However, PRB-FFF-regulated genes showed no significant enrichment for any of these hallmark gene sets due to attenuated gene regulatory activity. For example, more than 80% of PR regulated genes (padj < 0.05) in HYPOXIA hallmark were hypo-regulated by PRB-FFF (Figure 3B, Supplementary File S2). These hypo-regulated genes include *DUSP1*, *F3*, *CCN1*, *BIRC3*, and *STAT5A*, which are involved in various aspects of hypoxic response.

Cellular hypoxia is involved in metabolic adaptation and cell survival [31,32]. The progestin-regulated hypoxia pathway is associated with mitochondria-mediated caspase-independent apoptosis in MCF-7 cells [33]. *BNIP3* and *BNIP3L* are target genes of PRB, and the proteins form pores on the mitochondrial outer membrane and sequester anti-apoptotic proteins such as BCL-2 and BCL-XL to induce apoptosis. R5020-induced expression of *BNIP3L* was lower by 30% by PRB-FFF compared to PRB (0.7 vs. 1.05 in log2fold), and this effect is also reflected at the protein level after 96 h and 72 h + 48 h R5020 treatment (Figure 3C). Although regulation of *BNIP3* expression was not affected by AF1 mutations, R5020 failed to increase BNIP3 protein in PRB-FFF cells, likely due to weakened regulation of other components in the hypoxia pathway (Figure 3C). Increased permeability of mitochondrial membrane causes release of DNA nuclease ENDOG and proteolytic enzyme HtrA2/Omi from intermembrane space, leading to DNA fragmentation and degradation of Inhibitor of Apoptosis Proteins (IAPs) [34,35,36]. ENDOG and HtrA2/Omi were also upregulated in PRB and PRB-QQQ cells as players of caspase-independent apoptosis, but not in PRB-FFF cells, in accordance with reduced hypoxic and apoptotic response.

#### 2.3.3. AF1 Is Required for Gene Regulation for the Initial Cell Cycle Acceleration

Since PRB-FFF was unable to induce the initial phase of cell cycle acceleration in response to R5020, we asked whether this loss of activity is due to a loss of activity in the regulation of cell cycle genes. Figure 3D shows that treatment with R5020 for 6 h upregulated cyclins (*CCND1*, *CCNE2*) and other cell cycle-promoting proteins, such as E2F transcription factors (*E2F1*, *E2F4*, *E2F6*), *MYC*, and growth signaling proteins *EGF*, *EGFR*, and *AKT2* in PRB cells. R5020 also downregulated CDK inhibitors, *CDKN1A* and *CDKN2B*, in PRB cells. Whereas most of these genes were similarly regulated in PRB-QQQ cells, the regulation was significantly attenuated for most genes in PRB-FFF cells (Figure 3D). Furthermore, PRB-induced upregulation of MYC target genes was also evidently attenuated by AF1-FFF mutation. The data support the notion that AF1 is important in gene regulation for the initial phase of cell cycle acceleration in the biphasic effect of progestin.

### 2.4. Different Mechanisms of Ligand-Independent and Ligand-Dependent Gene Regulation

**Figure 4:**
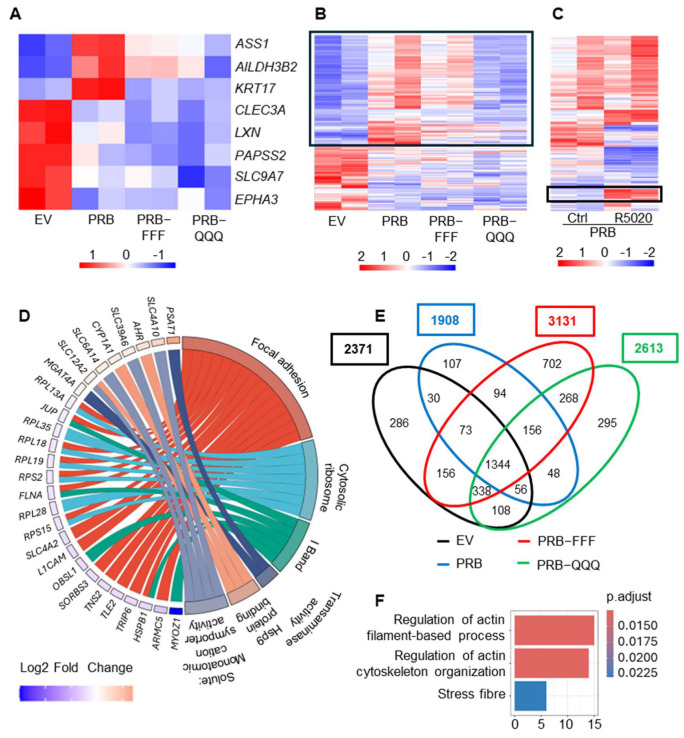
**AF1 is required for ligand-independent gene activation but not suppression by PRB.** (**A**) List of ligand-independent PR-regulated genes with padj < 0.05. (**B**) List of ligand-independent PR-regulated genes with *p*-value < 0.01. Genes in black box highlight reduced regulation by PRB-QQQ compared to PRB. (**C**) Heatmap showing that genes upregulated by unliganded PRB are not upregulated by R5020. Black box highlights the subset of genes regulated by R5020. (**D**) Chord diagram showing unliganded PRB down-regulated genes for focal adhesion and protein synthesis, and upregulated genes for metabolic adaptation and stress response. (**E**) Venn diagram indicates the overlapping of genes significantly regulated by E2 in EV, PRB, PRB-FFF and PRB-QQQ cells (padj < 0.05). (**F**) Unliganded PRB exerts repressive effects on a subset of estrogen-regulated genes involved in actin cytoskeleton dynamics.

AF1 was initially discovered as a ligand-independent activation domain in gene reporter assays. Studies have shown that PR regulates gene expression in a ligand-independent manner through phosphorylation by growth factor-initiated signaling [37,38,39]. Comparison between vector-transfected cells (EV cells) and PRB cells revealed eight genes regulated by unliganded PR based on padj < 0.05. Both AF1-FFF and AF1-QQQ mutations diminished the ligand-independent gene upregulation but had no effect on gene downregulation (Figure 4A).

The top three upregulated genes by unliganded PRB are *ASS1*, *ALDH3B2* and *KRT17* and serve distinct functions. *ASS1* (Argininosuccinate synthetase) catalyses the formation of argininosuccinate from citrulline and aspartate, a key step in the urea cycle and in the synthesis of arginine. *ASS1* is upregulated in response to DNA damage and halts cell-cycle progression by limiting nucleotide synthesis and transcription of a subset of p53-regulated genes [40]. *ALDH3B2* is an aldehyde dehydrogenase for aldehyde detoxification and has been reported to promote the growth and invasion of cholangiocarcinoma [41]. *KRT17* is a component of the intermediate filament, and its high expression is associated with idiopathic pulmonary fibrosis [42]. The ligand-independent upregulation of these three genes indicates that PRB influences these processes.

To have a deeper understanding of AF1 involvement in these effects, we expanded the ligand-independent gene set based on a less stringent *p*-value cutoff (*p* < 0.01 instead of padj < 0.05), and this yielded 232 ligand-independently regulated genes (Figure 4B, Supplementary File S3). In contrast to their hyper- and hypoactivity in response to R5020, the AF1-QQQ mutation largely abolished the upregulation of genes by unliganded PR, but the AF1-FFF mutation had little effect (Figure 4B, boxed region). Furthermore, no notable effect of AF1 mutations was observed for the downregulated genes. Hence, these ligand-independent upregulated genes are AF1-regulated, but the downregulated genes are not AF1-regulated. This suggests that the mechanisms of ligand-independent and ligand-dependent gene regulation are different because of the different effects of AF1 mutations on their regulation.

Ligand-independent activity of PR was initially discovered to exhibit activity in the PRE reporter gene assay, which is routinely used to demonstrate ligand-induced activity of PR and of nuclear receptors in general. Similarly, current understanding of ligand-independent activity of PR through growth factor-initiated signaling has also been based on regulation of known ligand-regulated PR target genes or response in PRE reporter gene assays. For example, the expression of PR target genes *HB-EGF*, *IRS-1*, and *STC1* was used to demonstrate that PR sumoylation represses protein kinases (MAPK, CDK2) mediated ligand-independent activity of PR [43]. These genes are regulated by PR in response to ligand but also regulated by protein kinases through PR phosphorylation in the absence of ligand [44]. However, most ligand-independent regulated genes identified from our RNA-Seq analysis were not significantly regulated by R5020. In fact, only 18 of the 232 (8%) ligand-independently regulated PR target genes were regulated by R5020 (Figure 4C, boxed region). Furthermore, the 18 genes were upregulated by R5020 but downregulated by unliganded PRB. Therefore, this first genome-wide profiling of ligand-independent activity of PRB and AF1 suggests that the ligand-independently regulated genes are generally not ligand-regulated and likely to serve different biological purposes.

Gene Ontology analysis of the 232 ligand-independent regulated genes shows significant upregulation of genes for Hsp90 protein binding (*SLC12A2*, *CYP1A1*, *AHR*), genes for solute monoatomic cation symporter activity (*SLC12A2*, *SLC6A14*, *SLC39A6*, *SLC4A10*) and transaminase activity (*PSAT1*, *MGAT4A*), which reflect enhanced metabolic adaptation and stress response (Figure 4D). Genes in GO terms for focal adhesion, cytosolic ribosomes, and I band were all downregulated, suggesting ligand-independent repression of cellular activities, including focal adhesion and protein synthesis.

We also evaluated whether PR exerts a ligand-independent effect on estrogen-regulated gene expression, and whether the effect involves the activity of AF1. After treatment with E2 for 6 h, 2391, 1908, 3131 and 2613 genes were significantly regulated in EV, PRB, PRB-FFF and PRB-QQQ cells, respectively (padj < 0.05, Figure 4E). A total of 1344 genes were significantly regulated in all four cell lines, while 286 genes, 107 genes, 702 genes and 295 genes were uniquely regulated (padj < 0.05) by EV, PRB, PRB-FFF and PRB-QQQ, respectively. E2 regulated fewer genes in PRB cells than in EV cells, with 11.4% (271) of the genes being less regulated by more than 20% in PRB cells based on fold change. Thus, PRB exerts some repressive effects on a subset of estrogen-regulated genes independent of R5020. GO terms enriched in this gene set are actin filament-based process, actin cytoskeleton organization and stress fibers (Figure 4F).

On the other hand, AF1 mutations did not show a clear effect on how unliganded PRB influences E2-induced gene expression, despite 40% and 27% more genes being significantly regulated by E2 in PRB-FFF and PRB-QQQ cells, respectively. Comparison of uniquely regulated genes by mutants with PRB in heatmaps shows that genes significantly regulated by AF1 mutants were also regulated by PRB, albeit statistically insignificant due to higher or lower basal levels in untreated cells (Supplementary Figure S4). It is thus likely that AF1 subtly modulates estrogen-regulated gene expression in a ligand-independent manner.

### 2.5. The Impact of AF1-FFF and AF1-QQQ Mutations on Synergistic and Antagonistic Activity Between E2 and R5020 Is Largely Due to Its Influence on R5020 Response

**Figure 5:**
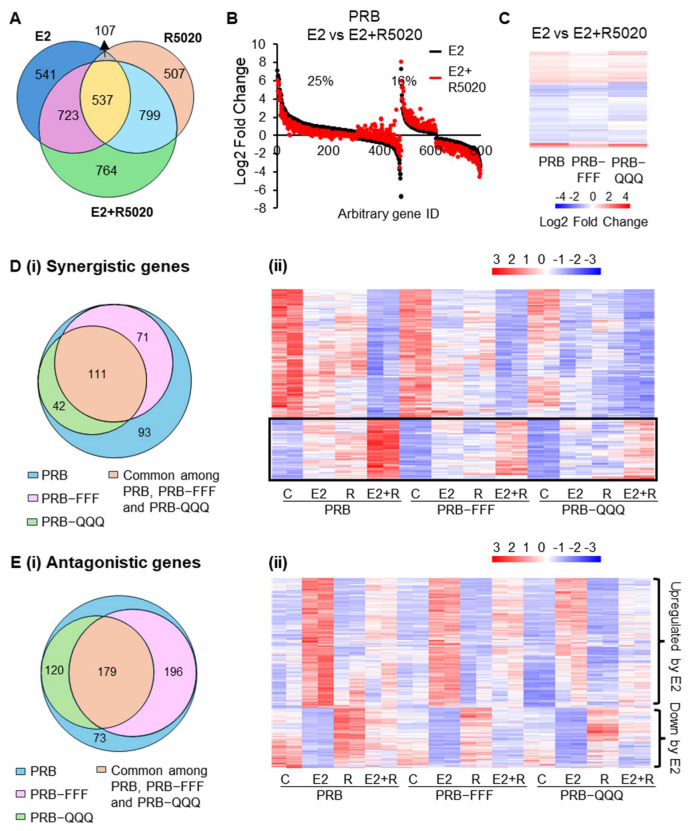
**Influence of AF1 on synergistic and antagonistic activity between E2 and R5020 is largely due to its involvement in R5020 Response.** (**A**) Venn diagram indicates the overlap of genes significantly regulated by E2, R5020 and E2 + R5020 in PRB cells (padj < 0.05). (**B**) A total of 41% of genes in PRB cells show more than 20% difference in regulation when treated with E2 + R5020 compared to E2 alone, and 25% of these show less regulation by PRB. The fold change values represent the ratio of E2 + R5020-treated to E2-treated in PRB cells. (**C**) AF1-FFF mutation attenuated the effect of R5020 on E2-regulated genes. The fold change values represent the ratio of E2 + R5020-treated to E2-treated within each respective cell line. (**D**) Synergistic genes identified in PRB cells and the comparison with PRB-FFF and PRB-QQQ cells. (**i**) Venn diagram shows overlap of synergistic genes between PRB, PRB-FFF and PRB-QQQ cells. (**ii**) Heatmap of synergistic genes. Boxed region highlights genes for which AF1 mutations attenuate the synergistic effect. C: Control; E2: Estradiol; R: R5020; E2 + R: Estradiol and R5020. (**E**) Antagonistic gene identified in PRB cells and the comparison to PRB-FFF and PRB-QQQ cells. (**i**) Venn diagram indicates the number of overlapping antagonistic genes in PRB, PRB-FFF and PRB-QQQ cells. (**ii**) Heatmap of antagonistic genes. C: Control; E2: Estradiol; R: R5020; E2 + R: Estradiol and R5020.

Since progesterone is known to exert synergistic and antagonistic effects with estrogen on gene regulation, we investigated whether AF1 is involved in this functional interplay. DESeq2 analysis showed that 2823 genes were significantly regulated in PRB cells in response to combined E2 + R5020 treatment, as compared to 1908 genes in response to E2 and 1950 genes in response to R5020 alone (Figure 5A). A total of 41% of E2-regulated genes in PRB cells were differentially regulated by greater than 20% in response to E2 + R5020, suggesting co-regulation by E2 and R5020 (Figure 5B). This is consistent with known functional interplay between PR and ERα [45,46]. AF1-FFF mutation attenuated the effect of R5020 on some of the E2-regulated genes, resulting mainly in less up- or downregulation in PRB-FFF cells compared to PRB cells (Figure 5C). On the other hand, fold changes between E2 + R5020 and E2-treated PRB-QQQ cells can be higher or lower than those in PRB cells, depending on their response to R5020.

To define genes synergistically regulated by E2 and R5020, E2 + R5020-regulated genes must be significantly different over vehicle-treated, E2-treated and R5020-treated alone. Additionally, the magnitude of gene regulation by E2 + R5020 must be greater by 20% than that by R5020 or E2 alone. This yielded 317 genes from PRB cells (Figure 5D(i), Supplementary File S4). Heatmap of the 317 genes shows that AF1 mutations attenuated the synergistic effect on upregulated genes (Figure 5D(ii), boxed region) largely due to less upregulation in response to R5020 or E2 in PRB-FFF and PRB-QQQ cells. Notable synergistically regulated genes include *E2F6* and *TGFA*, which promote cell growth, and *CD44*, a cancer stem cell marker. This is not surprising considering that E2 or R5020 alone induced a mitogenic program. But prolonged treatment of R5020 induced cell cycle arrest.

Antagonistically regulated genes are defined as E2-regulated genes whose magnitude of regulation by E2 + R5020 was at least 20% less than that by E2 alone or exhibited in an opposite regulatory direction. A total of 568 E2 target genes were thus listed as antagonistically regulated by R5020 in PRB cells (Figure 5E(i), Supplementary File S5). For E2-upregulated genes, R5020 alone did not have a significant effect but antagonized E2-induced upregulation (Figure 5E(ii)). Furthermore, AF1 mutations did not influence the antagonist effect of R5020 notably. On the other hand, E2-downregulated genes were upregulated by R5020, and this reversed E2-induced downregulation when R5020 was added together with E2. Although AF1 mutations attenuated the upregulation by R5020 in some of the genes, PRB-FFF and PRB-QQQ were still able to attenuate E2-induced downregulation of the genes in response to R5020 (Figure 5E(ii). Taken together, AF1-FFF and AF1-QQQ mutations affected synergistic and antagonistic activity between E2 and R5020, largely based on their effect on R5020 response.

### 2.6. PRB-FFF Exhibits Greater Genome-Wide Binding than PRB and PRB-QQQ

#### 2.6.1. ChIP-Seq Analysis Detected More PRB-FFF Binding Sites than PRB

**Figure 6:**
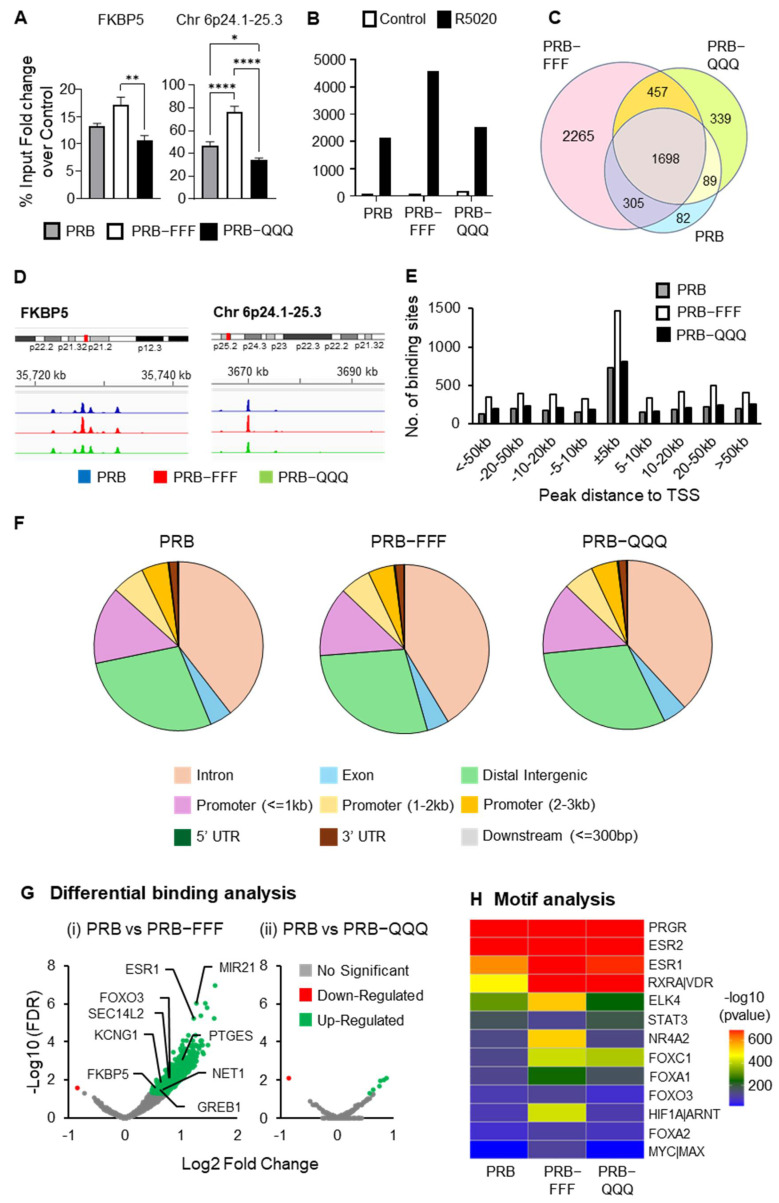
**PRB-FFF exhibits greater genome-wide binding than PRB.** (**A**) ChIP-qPCR analysis of PR target genes showing greater enhancer binding of PRB-FFF in response to R5020 treatment. Data are expressed as percentage of input, calculated as the ratio of signal for R5020-treated to vehicle-treated cells. Asterisks indicate statistical significance between the respective cell lines (* *p* < 0.05, ** *p* < 0.01, **** *p* < 0.0001). (**B**) Total number of consensus peaks identified from biological triplicates (*n* = 3) in PRB, PRB-FFF, and PRB-QQQ cells. Cells were treated with vehicle (EtOH) or 10 nM R5020 for 1 h prior to ChIP-Seq analysis. (**C**) Venn diagram shows the overlap of R5020-induced peaks among PRB (blue), PRB-FFF (pink) and PRB-QQQ (green) cells. (**D**) Genome browser tracks illustrate the binding peaks at representative PR target genes. (**E**) Distribution of PR binding sites relative to their nearest TSS. Highest enrichment occurs at ±5 kb of the TSS across all cell lines, with PRB-FFF exhibiting the highest number of binding sites. (**F**) Distributions of PR peaks by PRB, PRB-FFF and PRB-QQQ in different gene regions are highly similar. (**G**) Volcano plots show significantly more differential binding by PRB-FFF but not by PRB-QQQ compared to PRB. The fold change values represent the ratio of R5020-treated PRB-FFF to PRB. (**H**) Relative enrichment of transcription factor binding motifs associated with PR peaks in PRB, PRB-FFF and PRB-QQQ cells. Negative logarithm of *p*-value is shown, blue indicates non-significant enrichment, and yellow to red indicates increasing significance.

We reported that AF1-FFF mutation enhanced the ligand-induced interaction between AF1/NTD and SRC-1, and between AF1/NTD and AF2/LBD [19]. We proposed a tripartite model in which the transient and calibrated interactions among AF1, AF2 and coregulators are important for a productive enhancer and dynamic cycling of the transcription complex. We hypothesize that a more stable interaction due to the AF1-FFF mutation results in prolonged enhancer occupancy and a slower transcription rate. The prolonged enhancer occupancy would mean higher levels of PRB-FFF chromatin binding. Indeed, ChIP-qPCR showed that levels of PRB-FFF binding to the enhancer regions of FKBP5 and Chr 6p24.1-25.3 were higher than those of PRB and PRB-QQQ (Figure 6A).

A ChIP-Seq experiment was performed using three independent biological replicates per condition. Principal component analysis (PCA) demonstrated tight clustering of biological triplicates and clear separation between control and R5020-treated samples, indicating strong treatment-dependent effects (Supplementary Figure S5A). A consensus peak set derived from biological triplicates was subsequently used for all downstream analyses. ChIP-Seq analysis revealed 2177 PRB binding peaks. The number of binding peaks of PRB-FFF is more than twice that of PRB (4725 vs. 2174), whereas the number of binding peaks of PRB-QQQ (2583) is 18% higher (Figure 6B). About 78% and 74% of PRB binding peaks are shared with PRB-FFF and PRB-QQQ, respectively (Figure 6C). Although the number of PRB binding peaks is considerably lower than the number of binding peaks in T47D cells [47], commonly known PR binding sites on target genes such as *FKBP5*, *Chr 6p24.1-25.3* (Figure 6D), *HSD11B2*, *NET1* and *STAT5A* (Supplementary Figure S5B) are well-represented in our data.

PR binding is approximately 5-fold higher within the 5 kb of the TSS compared to other regions, regardless of the cell line (Figure 6E). Consistently, higher PRB-FFF binding peaks are seen in regions −5 kb to +5 kb from TSS and cross all regions of chromosomes (Figure 6E, Supplementary Figure S5C), although there are many fewer binding peaks on chromosome X (Supplementary Figure S5C). On the other hand, binding distributions across promoter regions, introns, intergenic regions, etc., were similar between PRB and AF1 mutants (Figure 6F).

#### 2.6.2. PRB-FFF Binding to Transcription Factor Motifs Is Significantly Higher than PRB

Differential binding analysis showed 902 differentially bound regions between R5020-treated PRB-FFF and PRB (FDR < 0.05), of which 901 peaks show greater binding by PRB-FFF than by PRB (Figure 6G(i)). These include known PR target genes *ESR1*, *MIR21*, *FOXO3* and *PTEGS* with greater PRB-FFF binding. In contrast, PRB-QQQ showed only a few genes with significant differential binding compared to PRB (Figure 6G(ii)). Higher levels of chromatin binding by PRB-FFF are plausibly due to its stronger interaction with SRC-1 and AF2 [19], which could slow down transcription complex disassembly. Hence, the data support the notion that AF1 is involved in modulating the dynamics of PR-chromatin interaction.

Expectedly, motif analysis using MEME Suite revealed that the topmost enriched binding motif of PRB, PRB-FFF and PRB-QQQ is PR consensus motif GGTACANNNT-GTTCT (Supplementary Figure S5D). While this identified motif deviates from the perfectly palindromic canonical PRE at the first half-site, it aligns with common PR consensus sequences reported in genomic databases and literature [48]. Other enriched motifs belong to the zinc finger family of proteins whose functions are not well-characterized (Supplementary Figure S5D). Because our dataset contains a relatively low number of peaks, yielding only a limited number of motifs, we employed another motif discovery tool, MDSeqPos to further identify the enriched motifs. The top three enriched motifs were for PGR, ESR1 and ESR2 binding (Figure 6H). This is consistent with the understanding that PR is often associated with ESR and recruited to its binding regions in response to progestin [45]. In addition, R5020-induced PRB-chromatin binding sites were also significantly enriched with motifs for RXR-VDR, ELK4, NR4A2, FOXA1 and HIF1A. PR binding to these motifs likely occurs through association with these transcription factors or in a process known as ‘tethering’ [49]. PRB-FFF binding to these motifs is significantly more enriched than PRB and PRB-QQQ based on *p*-values. The RXR-VDR heterodimer is a nuclear receptor complex consisting of the Vitamin D Receptor (VDR) and Retinoid X Receptor (RXR). It acts as a ligand-activated transcription factor, where RXR is a required partner for VDR to bind DNA in response to vitamin D3 and regulate gene transcription. Based on our literature search, this is the first evidence of PR tethering to RXR-VDR, thereby regulating their target gene expression. ELK4 is an ETS domain-containing protein and is also reported to be enriched at binding sites for ERα/PR complexes [50]. NR4A2 is a nuclear orphan receptor, and its known target gene, *BNDF*, is also a PR target gene in breast cancer cells and in neuronal tissue [25,50]. Our study provides the first evidence that PRB regulates NR4A2 target genes through a tethering mechanism. FOXA1 is a pioneer factor that allows for ERα and PR access to target chromatin binding sites [51]. Its binding sites are known to be overrepresented in PRB binding regions [52]. Since these motifs are significantly more enriched in PRB-FFF binding sites, AF1 likely plays a role in modulating dynamics of PR tethering with ELK4, NR4A2, FOXA1 and HIF1A.

We next performed integrated Chip-Seq and RNA-Seq data analysis to determine if greater PRB-FFF-chromatin binding is associated with attenuated gene regulation. Of the 901 sites showing increased occupancy by the PRB-FFF, 306 (34%) were associated with genes significantly regulated in PRB cells. A total of 83% of these PRB-target genes (253 of 306) exhibited attenuated regulation by PRB-FFF. Additionally, 104 genes of the 306 genes (34%) were no longer significantly regulated by PRB-FFF (padj > 0.05), while another 149 (49%) showed diminished fold-changes compared to PRB. These data indicate that while the AF1-FFF mutation enhances genomic binding, it effectively uncouples DNA occupancy with functional transactivation, hindering the assembly of a dynamic, productive transcriptional complex.

## 3. Discussion

The current study evaluated the roles of PR AF1 in progestin regulation of cellular activity and gene expression in breast cancer cells. It made three lines of significant findings. First, AF1 is involved in progestin regulation of cell growth and apoptosis. Loss-of-function mutant PRB-FFF exhibited marked impairment in these activities. It failed to upregulate genes for proliferation to accelerate the initial cell cycle progression in the biphasic effect of progestin. Second, AF1 activity is gene-specific. AF1 mutations affected the regulation of approximately half of PR target genes. Furthermore, ligand-dependent and independent activities of AF1 are mediated through distinct mechanisms because AF1-FFF and AF1-QQQ mutations affected ligand-dependent and independent activities differently. Importantly, PR AF1 is involved in modulating PR–chromatin interaction as the hypoactive PRB-FFF shows more enhancer binding than PRB, likely due to stronger interaction with SRC-1 and AF2, as was previously reported [19]. Taken together, PR AF1 is important for the core activities of liganded PR in regulating growth, and it is involved in the modulation of PR-chromatin interactions through cofactor interactions.

AF1 was originally discovered as a ligand-independent activation domain of chick PR using reporter gene assays [12]. Ligand-independent activity of PR and steroid hormone receptors is traditionally defined by activity in a reporter gene assay or interaction with transcription coactivators [53,54]. It may be influenced by the cellular context or expression of coregulators such as PRMT6 [55]. This study made two novel observations from RNA-Seq analysis of ligand-independent activity. First, most genes regulated by unliganded PR are not regulated by R5020. This suggests that ligand-independent activity of PR has a distinct biological role. Second, the property of amino acid required for ligand-induced activity of AF1 is different from the ligand-independent activity because the loss-of-function mutant PRB-FFF mutations had little effect on ligand-independent gene regulation, whereas the PRB-QQQ mutant, which was hyperactive in response to progestin, lost most of the ligand-independent activity. It is plausible that the ligand-independent activity of PR involves AF1 with KKR methylation because the PRB-FFF mutant with increased local bulkiness and hydrophobicity works well for the ligand-independent gene regulation.

The dynamics of nuclear receptor-chromatin interactions have a direct impact on transcription rate [56,57]. It is influenced by binding kinetics with coregulators, chaperones, general transcription factors, as well as chromatin structure [58,59,60]. We propose that PR AF1 is involved in modulating the dynamics of PR–chromatin interaction through its interaction with coregulators and the general transcription machinery, based on the following evidence. First, loss-of-function mutant PRB-FFF exhibits significantly stronger interaction with SRC-1 and AF2, and this is associated with markedly slower receptor activation kinetics of the mutant [19]. Second, these stronger interactions are associated with more chromatin occupancy as indicated by more binding of the PRB-FFF mutant than the PRB in ChIP-Seq analysis. More chromatin binding in this case is likely due to a slower rate of dissociation resulting from more stable binding with its partner proteins, and hence slower activation kinetics. Additionally, modulation of PR–chromatin interaction by AF1 could also be regulated by post-translational modifications. We have shown that K464, K481 and R492 are monomethylated [19,61]. The bulky and hydrophobic phenylalanine (F) mimics the increased bulkiness and hydrophobicity of methylation. Hence, AF1 methylation could stabilize its interaction with partners such as SRC-1 and AF2 to reduce the disassembly kinetics of the PR transcription complex.

PR Cryo-EM structure shows direct interaction of AF1 with SRC-2 [16]. AF1 also binds to TATA box binding protein (TBP), and this binding alters the structure and mobility of AF2, although there is no direct interaction between AF2 and TBP [62]. Since AF1 interacts with both enhancer-associated coactivators and promoter-associated TBP, it is tempting to speculate that AF1 facilitates enhancer-promoter looping, in which the enhancer is brought in contact with its target promoter through coactivators and general transcription factors [63]. This notion is also supported by the evidence that TATA-binding protein (TBP) binding to glucocorticoid receptor AF1 induces AF1 folding and facilitates its interaction with SRC-1 [17], which in turn brings the promoter and enhancer together for transcription regulation.

To estimate whether AF1 methylation and AF1-FFF mutation indeed affect binding affinities between SRC-1 and AF1 [2,3], we used AlphaFold3 [64] to predict ternary assemblies of AF1, the conserved glutamine-rich region (aa 1053 −1123) of SRC-1 (where AF1 binds) [65], and TBP, and subsequently used PRODIGY to estimate the binding affinities [66,67]. Low ipTM scores of the predicted assemblies are expected due to the intrinsically disordered nature of AF1. Consistent with our hypothesis, the binding affinity of AF1 with monomethylation at KKR had a much higher binding affinity with SRC-1 (Kd = 9.2 × 10^−10^ M) than the unmethylated AF1 (Kd = 1.1 × 10^−6^ M). The methylation-mimetic PRB-FFF mutant also exhibited a higher binding affinity (Kd = 5.6 × 10^−10^ M) (Supplementary Figure S6). These effects are consistent with increased hydrophobic and steric stabilization of the AF1–SRC-1 interface by monomethylation or methylation mimic mutation. Structural analysis indicates that methylation of the KKR residues in the wild-type AF1 (PRB) does not directly participate in the interaction interface but instead promotes a closer spatial approximation of the peptide chains, indirectly stabilizing the complex. In contrast, the AF1-FFF mutation introduces direct interface contacts, with K464F contributing to interactions with SRC-1 (Supplementary Figure S6, arrowed). Predictions of hydrogen bonds (Supplementary Figure S6B) and hydrophobic contacts (Supplementary Figure S6C) reveal an increase in interfacial interactions from wild-type AF1 to methylated KKR, with the strongest interaction for the PRB-FFF mutant. This in silico prediction is consistent with results from co-immunoprecipitation and mammalian two-hybrid assay that showed greater interaction between SRC-1 and PRB-FFF than the wild-type AF1 (PRB) [19]. In the cellular context, methylation-driven stabilization of the interaction of an intrinsically disordered AF1 with coregulators such as SRC-1 could moderate transcription complex dynamics.

In summary, this study demonstrated that AF1 is required for the regulation of about 60% of PR target genes, including those for cell proliferation, hypoxia, and TNFA signaling. This study also suggests that AF1 is involved in modulating ligand-induced PR–chromatin interaction through AF1 monomethylation, which stabilizes the interaction with coregulators such as SRC-1. Since AF1 of steroid hormone receptors is a promising target for cancer treatment [22,23,24], the knowledge of crucial roles of PR AF1 in the regulation of breast cancer cell proliferation and apoptosis indicates that PR AF1 may be targeted with small molecules to achieve desired therapeutic outcomes.

## 4. Materials and Methods

### 4.1. Cell Culture

The MCF-7 cell line was sourced from the American Type Culture Collection (ATCC). Cells were cultured in Dulbecco’s Modified Eagle’s Medium (DMEM) with phenol red (08488-55, Nacalai Tesque, Kyoto, Japan) supplemented with 7.5% fetal calf serum (FCS) and 2 mM L-glutamine (Hyclone, Logan, UT, USA). Cultures were maintained at 37 °C in a humidified incubator with 5% CO_2_ in air.

### 4.2. Gene Cloning

Site-directed mutagenesis was employed to generate PR AF1 K464F_K481F_R492F and K464Q_K481Q_R492Q mutants as previously reported [19]. PRB cDNA was cloned into the pLVX-Puro vector using XhoI and Xbal restriction enzyme sites. Plasmid vectors carrying PRB and mutant cDNAs were designated as PRB, PRB-FFF and PRB-QQQ. An empty vector (EV) served as control. All constructs were verified by DNA sequencing.

### 4.3. Generation of MCF-7 Stable Cell Lines Overexpressing PRB and PR AF1 Mutants

The MCF-7 cells were seeded at 1 × 10^5^ cells per well in a 6-well plate. Then, 24 h later, cells were transduced with lentiviruses carrying PRB or AF1 mutant cDNA at a Multiplicity of Infection (MOI) of 2 in the presence of polybrene. The cells were then centrifuged for 90 min at 30 °C and 1000× *g* with maximum acceleration and minimum deceleration without braking. Cells were then incubated for 48 h before being transferred to a 100 mm culture dish and cultured in selection medium containing 2.5 ug/mL puromycin. A second selection was performed five days later, after allowing the cells to recover for one week. The resulting stable MCF-7 cell lines expressing PRB, PRB-FFF and PRB-QQQ and empty vector EV were subsequently maintained in antibiotic-free DMEM, and all experiments were conducted within 10 passages. Stable PR overexpression in mutant cell lines was routinely verified via western blotting and immunofluorescence.

### 4.4. Hormone Treatment

Cells were cultured for 48 h in phenol-red-free DMEM supplemented with 5% dextran-charcoal-treated FCS (DCC-FCS) and 2 mM L-glutamine prior to hormone treatment. Hormone treatment includes promegestone (R5020), estrogen (17-beta estradiol) or a combination of both at designated concentrations in 0.01% ethanol in accordance with experimental design. Control groups received 0.01% ethanol alone.

### 4.5. Cell Cycle Analysis

Cells after 16 h and 48 h 10 nM R5020 treatment were detached by 0.05% trypsin and stained with propidium iodide (PI) in Vindelov’s cocktail [10 mM Tris-HCl (pH 8), 10 mM NaCl, 50 mg PI/L, 10 mg/L RNaseA, and 0.1% NP40]. Staining was performed in the dark for 30 min at 4 °C. Samples were then analyzed using a BD LSRFortessa™ X-20 flow cytometer (BD Biosciences, Franklin Lakes, NJ, USA) with excitation at 488 nm. Cell cycle distribution was determined using FlowJo software v10.8.1 (BD Biosciences). Data represent the mean of 12 replicates (*n* = 12) derived from four independent experiments.

### 4.6. Apoptosis Assay

Cells after 96 h and 72 + 48 h 10 nM R5020 treatment were detached by 0.05% trypsin and stained with anti-Annexin V antibody and propidium iodide (PI) using the Dead Cell Apoptosis Kit with Annexin V Alexa Fluor™ 488 & Propidium Iodide (Invitrogen, Carlsbad, CA, USA), according to the manufacturer’s protocol. Stained cells were analyzed using a BD LSRFortessa™ X-20 flow cytometer (BD Biosciences, Franklin Lakes, NJ, USA). Data represent the mean of 6 replicates (*n* = 6) derived from two independent experiments.

### 4.7. Cell Imaging and Fluorescent Microscopy

Cells were seeded at a density of 1 × 10^5^ cells per 35 mm dish, each with a glass coverslip in phenol-free DMEM supplemented with 5% DCC-FCS and 2 mM L-glutamine. After 48 h, cells were treated with either 0.01% ETOH (vehicle control) or 10 nM R5020. Cell images were captured using an Olympus phase contrast microscope (IX71; Olympus Corporation, Tokyo, Japan) after 24, 48, 72 and 96 h post-treatment.

For PR immunostaining, transduced cells grown on a glass coverslip were fixed with 3.7% formaldehyde and permeabilized with 0.2% Triton X-100, each for 10 min. Cells were then blocked for 1 h in blocking agent (2% FBS in PBS), followed by incubation with anti-PR antibody H190 (Santa Cruz Biotechnology Inc., Dallas, TX, USA, LOT number sc-7208) in a 1:200 dilution at 37 °C for 2 h. After washing, cells were incubated with Alexa Fluor™ 488-conjugated Goat anti-Rabbit IgG (Invitrogen, Carlsbad, CA, USA, Cat # A-11034) for 1 h. Coverslips were then mounted using Duolink^®^ In Situ Mounting Medium with DAPI (Sigma-Aldrich, St. Louis, MO, USA; Cat # DUO82040) for immunofluorescence imaging.

### 4.8. Protein Lysate Collection and Western Blotting Analysis

Cells were lysed using cold lysis buffer containing 50 mM HEPES-KOH (pH 7.5), 100 mM NaF, 150 mM NaCl and 1% Triton X-100. Lysates were collected from the supernatant after centrifugation at 12,000× *g* for 12 min. Proteins in total cell lysates were quantified and resolved by SDS-PAGE electrophoresis and transferred onto a nitrocellulose or PVDF membrane. Membranes were blocked with 5% skimmed milk or 2.5% BSA in Tris-buffered saline with Tween 20 (TBST), followed by overnight incubation with primary antibodies.

The primary antibodies used in the experiments are H190 Total PR (Santa Cruz Biotechnology Inc., Dallas, TX, USA, LOT number sc-7208), ERα (Santa Cruz Biotechnology Inc., Dallas, TX, USA, LOT number sc-8002), BNIP3 (Cell Signalling Technology, Danvers, MA, USA, #44060), BNIP3L/NIX (Cell Signalling Technology, Danvers, MA, USA, #12396), HTRA2/OMI (Cell Signalling Technology, Danvers, MA, USA, #9745), Endonuclease G (Cell Signalling Technology, Danvers, MA, USA, #4969) and GAPDH (Santa Cruz Biotechnology Inc., Dallas, TX, USA, LOT number sc-47724). Primary antibodies were used at a dilution of 1:1000, except for GAPDH, which was diluted at 1:10,000.

Horseradish peroxidase (HRP)-conjugated secondary antibodies, anti-mouse (Cell Signalling Technology, Danvers, MA, USA, #7076) and anti-rabbit (Cell Signalling Technology, Danvers, MA, USA, #7074) were used based on the host species of primary antibodies at dilution of 1:1000. Proteins detection was performed using Immobilon Western Chemiluminescent HRP substrate (Merck Millipore, Billerica, MA, USA) and ChemiDoc MP Imaging System (Bio-Rad Laboratories, Inc., Hercules, CA, USA). All Western blot experiments were performed in biological duplicates.

### 4.9. RNA Extraction

For transcriptomic analysis, biological duplicate samples (*n* = 2) were prepared for each condition. Following hormone treatment, cells cultured in 60-mm dishes were lysed in cold TRIzol reagent, and total RNA was isolated according to the manufacturer’s protocol (Life Technologies, Frederick, MD, USA). RNA was precipitated using isopropanol and pelleted at 12,000× *g*, 4 °C for 15 min. The pelleted RNA was washed twice with 75% ethanol prepared in DEPC-treated water, briefly air-dried and resuspended in DEPC-treated water.

Total RNA concentration and purity were assessed using a Nanodrop 2000 spectrophotometer (Thermo Fisher Scientific, Waltham, MA, USA). RNA integrity was further evaluated through agarose gel electrophoresis to check for RNA degradation.

### 4.10. RNA-Seq Analysis

Total RNA was treated with DNase I using the DNA-free™ DNA Removal Kit (Invitrogen, Carlsbad, CA, USA) to eliminate residual genomic DNA. DNA-free RNA samples were submitted to the Genome Institute of Singapore (Agency for Science, Technology and Research, Singapore) for library preparation and paired-end sequencing using the Illumina HiSeq4000 platform (Illumina, Inc., San Diego, CA, USA).

Sequencing read quality was assessed using *FastQC*. Transcription quantification was performed using the STAR aligners against the human reference genome GRCh38 (hg38) obtained from Ensembl. Transcript-level counts were summarized to gene-level counts using the R package *tximport* (v1.26.1; R version 4.2.2) [68]. Differential gene expression analysis was performed using *DESeq2* [69]. Genes with an adjusted *p*-value less than 0.05 (padj < 0.05) were considered as differentially expressed genes (DEGs). DESeq2-normalized counts data were used for subsequent downstream analysis. Heatmaps of differentially expressed genes were generated using the online platform SRplot (v2023; http://www.bioinformatics.com.cn/srplot, accessed 18 January 2025).

### 4.11. Gene Set Enrichment Analysis (GSEA)

GSEA was performed using the software downloaded from the official website https://www.gsea-msigdb.org/gsea/index.jsp (accessed 25 August 2024). Analyses were conducted using default parameters. An enriched gene set with a false discovery rate (FDR) of q < 0.25 was considered statistically significant. The hallmark gene sets from the Molecular Signature Database (MSigDB) were used, which represent the curated gene sets characterizing well-defined biological processes [70,71].

### 4.12. ChIP-Seq and ChIP-qPCR

For ChIP-seq analysis, biological triplicates (*n* = 3) were prepared for each cell line and treatment condition. Following treatment with vehicle or 10 nM R5020 for 1 h, cells were crosslinked with formaldehyde at a final concentration of 1% for 10 min at room temperature. The crosslinking was quenched by the addition of glycine to a final concentration of 0.125 M. Cells were then harvested, lysed, and subjected to sonication using Bioruptor Pico sonication system (Diagenode, Liège, Belgium) at 5 cycles of 30 s “ON” 30 s “OFF”. After centrifugation to remove cell debris, 1% of the lysate was reserved as an input control.

Chromatin immunoprecipitation was performed using anti-PR antibody H-190 (Santa Cruz Biotechnology Inc., Dallas, TX, USA, LOT number sc-7208). The lysate was incubated with 5 ug anti-PR antibody overnight at 4 °C. The protein–chromatin complex was captured using Pierce™ ChIP-grade Protein A/G Magnetic Beads (ThermoFisher Scientific, Cat. #26162). Chromatin was eluted, and crosslinks were reversed by treatment with RNase A and Proteinase K. Input samples and ChIP’ed DNA were purified using a QIAquick PCR Purification Kit (Qiagen, Hilden, Germany). DNA concentrations were quantified using a Qubit 3.0Fluorometer (ThermoFisher Scientific, Waltham, MA, USA). The samples were submitted to the Genome Institute of Singapore (Agency for Science, Technology and Research) for library preparation and paired-end sequencing using the Illumina HiSeq4000 platform.

ChIP Quantitative real-time PCR was carried out using KAPA SYBR FAST qPCR Master Mix (Kapa Biosystems, Wilmington, MA, USA) on an ABI Prism 7700 sequence detection system (Applied Biosystem, Foster City, CA, USA) based on the manufacturer’s protocol. Real-time PCR for each targeted gene was performed in duplicates. The fold enrichment of the target sequence in the immunoprecipitated (IP) fraction was calculated using the comparative Ct method, normalized to the input fraction and expressed relative to vehicle-treated controls. The primer sequence is listed as follows [18,48]:

FKBP5 FW: 5′-TAATAGAGGGGCGAGAAGGCAGA-3′

FKBP5 RV: 5′-GGTAAGTGGGTGTGCTCGCTCA-3′

CHR6P FW: 5′-TCAGGAACAGTACACGAACGA-3′

CHR6P RV: 5′-CTGGCTCATCTTTCAGCACA-3′

### 4.13. ChIP-Seq Analysis

Model-based analysis of ChIP-Seq version 2 (MACS2) was used to perform peak calling for ChIP-Seq data, following the default parameters [72]. Integrative Genome Viewer (IGV) was used to create a representative snapshot for the peaks called [73]. Diffbind (v3.8.4), a R Bioconductor package, was used to identify the peaks with differential binding between cell lines and treatment [74].

Binding sites were extended by 50 bp on both 5′ and 3′ ends, and the corresponding FASTA sequences were fetched using the Galaxy platform [75]. The sequences were scanned for motif matches using the MEME-ChIP suite [76]. Motif analysis was also performed with the Seqpos motif tool (v1.0.0; Cistrome) [77].

### 4.14. Statistical Analysis

All s tatistical analyses were performed using the Graphpad Prism 9 software. Data are expressed as mean ± SEM (standard error of the mean). The degree of statistical significance is indicated with asterisks (* *p* < 0.05, ** *p* < 0.01, *** *p* < 0.001, **** *p* < 0.0001).

## Data Availability

The RNA-Seq and ChIP-Seq data that support the findings in this study are available in the ArrayExpress database (http://www.ebi.ac.uk/arrayexpress (accessed on 27 October 2025) under accession numbers E-MTAB-16059 and E-MTAB-15999, respectively.

## Supplementary Materials

The following supporting information can be downloaded at: https://www.mdpi.com/article/10.3390/ijms27062916/s1.
