# Supplementary material for: Ligand-Dependent and -Independent Functions of Activation Function 1 of Progesterone Receptor in Genome-Wide Gene Regulation and in Cell Proliferation and Apoptosis of Breast Cancer Cells
Source: Int J Mol Sci. 2026 Mar 23;27(6):2916. doi: 10.3390/ijms27062916 (PMC13027182; doi:10.3390/ijms27062916)
Supplement: Supplementary file 1 [file ijms-27-02916-s001.zip › Supplementary Figure_Proofread.pptx]

## Slide 1
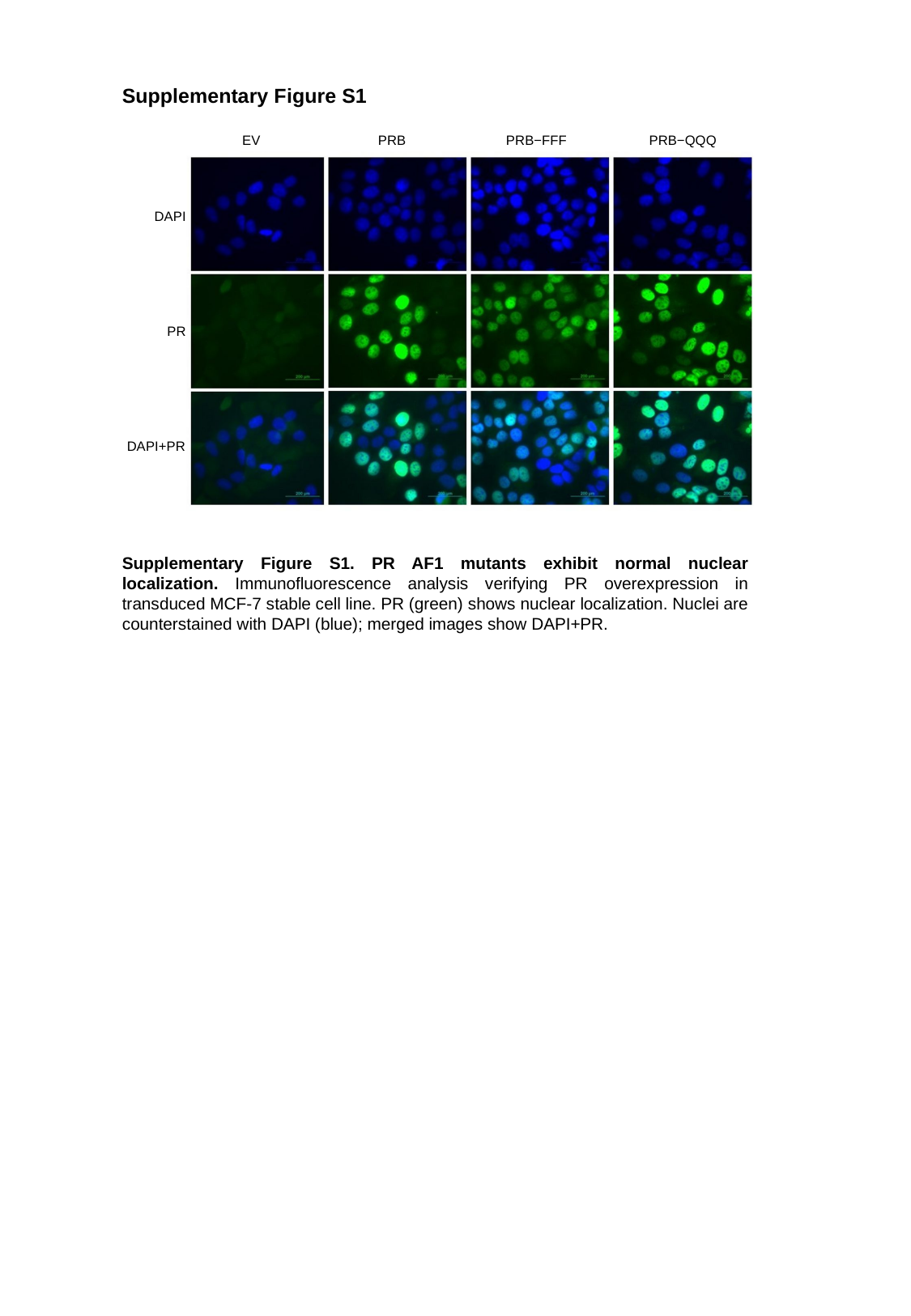

Supplementary Figure S1
EV
PRB
PRB−FFF
PRB−QQQ
DAPI
PR
DAPI+PR
Supplementary Figure S1. PR AF1 mutants exhibit normal nuclear localization. Immunofluorescence analysis verifying PR overexpression in transduced MCF-7 stable cell line. PR (green) shows nuclear localization. Nuclei are counterstained with DAPI (blue); merged images show DAPI+PR.

## Slide 2
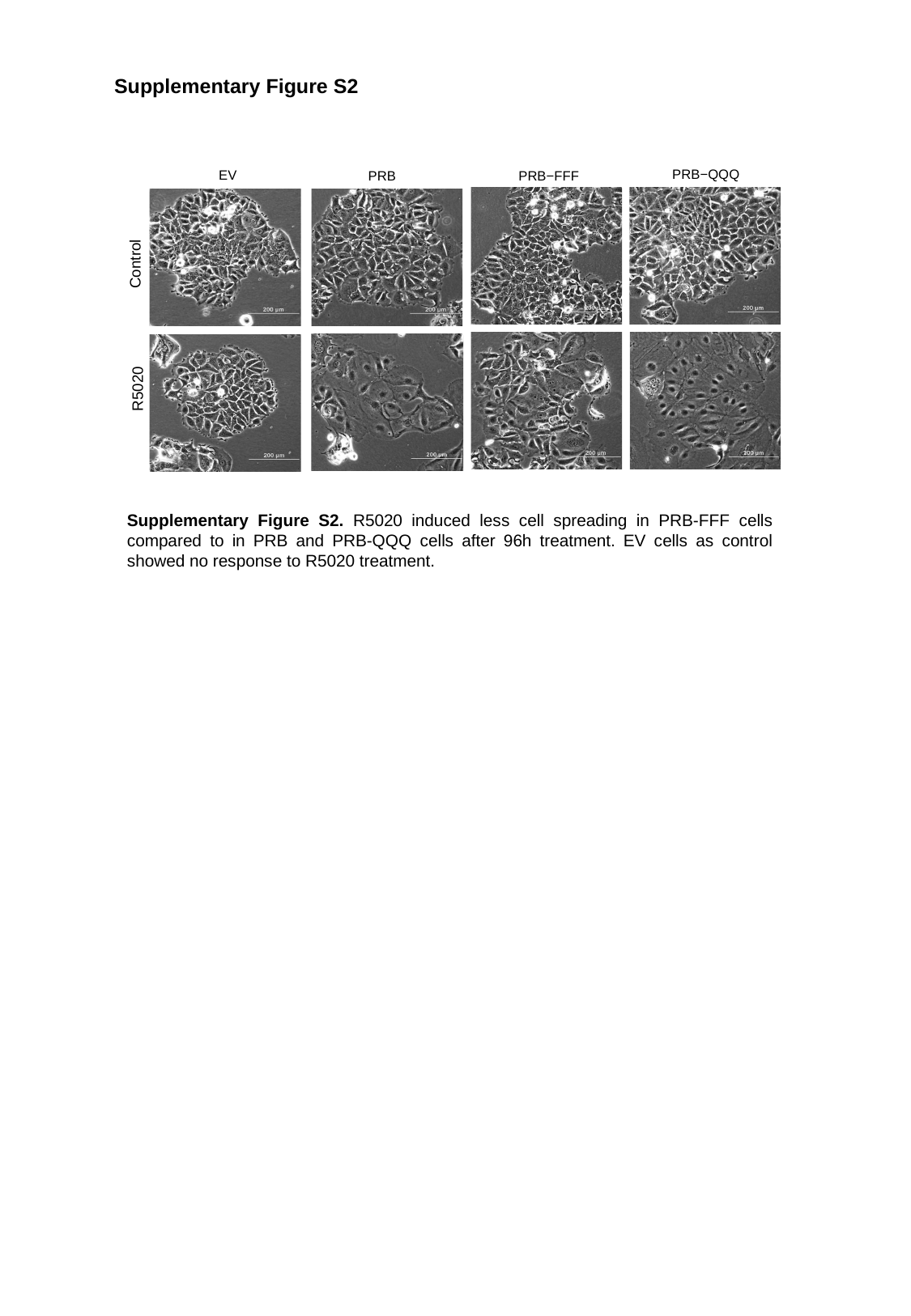

Supplementary Figure S2
PRB−QQQ
EV
PRB
PRB−FFF
Control
R5020
Supplementary Figure S2. R5020 induced less cell spreading in PRB-FFF cells compared to in PRB and PRB-QQQ cells after 96h treatment. EV cells as control showed no response to R5020 treatment.

## Slide 3
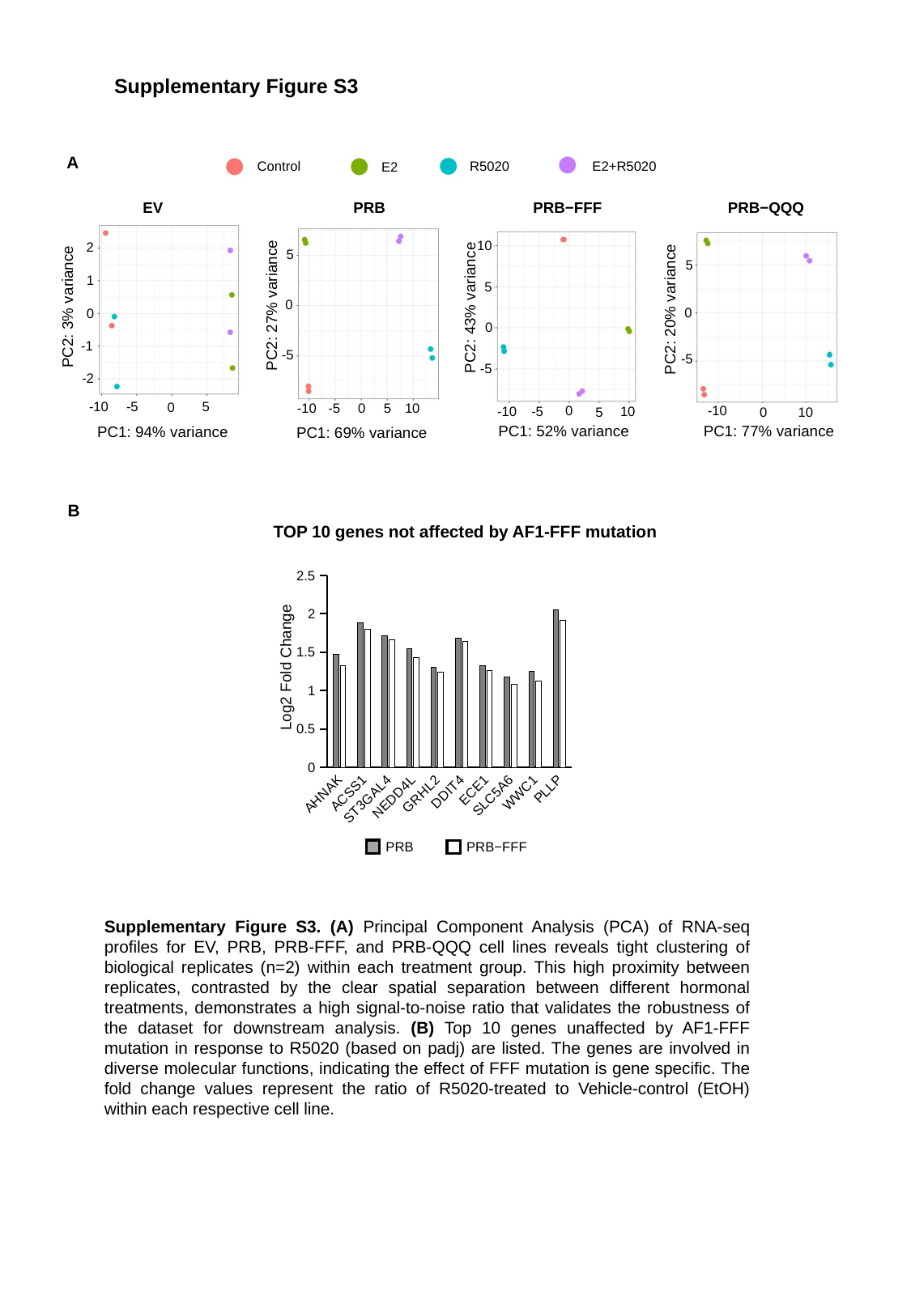

Supplementary Figure S3
A
Control
E2
R5020
E2+R5020
EV
2
1
PC2: 3% variance
0
-1
-2
5
-5
-10
0
PC1: 94% variance
PRB
5
0
PC2: 27% variance
-5
-10
-5
0
5
10
PC1: 69% variance
PRB−FFF
10
5
PC2: 43% variance
0
-5
0
10
-10
-5
5
PC1: 52% variance
PRB−QQQ
5
PC2: 20% variance
0
-5
-10
0
10
PC1: 77% variance
B
TOP 10 genes not affected by AF1-FFF mutation
[unsupported chart]
Log2 Fold Change
PRB
PRB−FFF
Supplementary Figure S3. (A) Principal Component Analysis (PCA) of RNA-seq profiles for EV, PRB, PRB-FFF, and PRB-QQQ cell lines reveals tight clustering of biological replicates (n=2) within each treatment group. This high proximity between replicates, contrasted by the clear spatial separation between different hormonal treatments, demonstrates a high signal-to-noise ratio that validates the robustness of the dataset for downstream analysis. (B) Top 10 genes unaffected by AF1-FFF mutation in response to R5020 (based on padj) are listed. The genes are involved in diverse molecular functions, indicating the effect of FFF mutation is gene specific. The fold change values represent the ratio of R5020-treated to Vehicle-control (EtOH) within each respective cell line.

## Slide 4
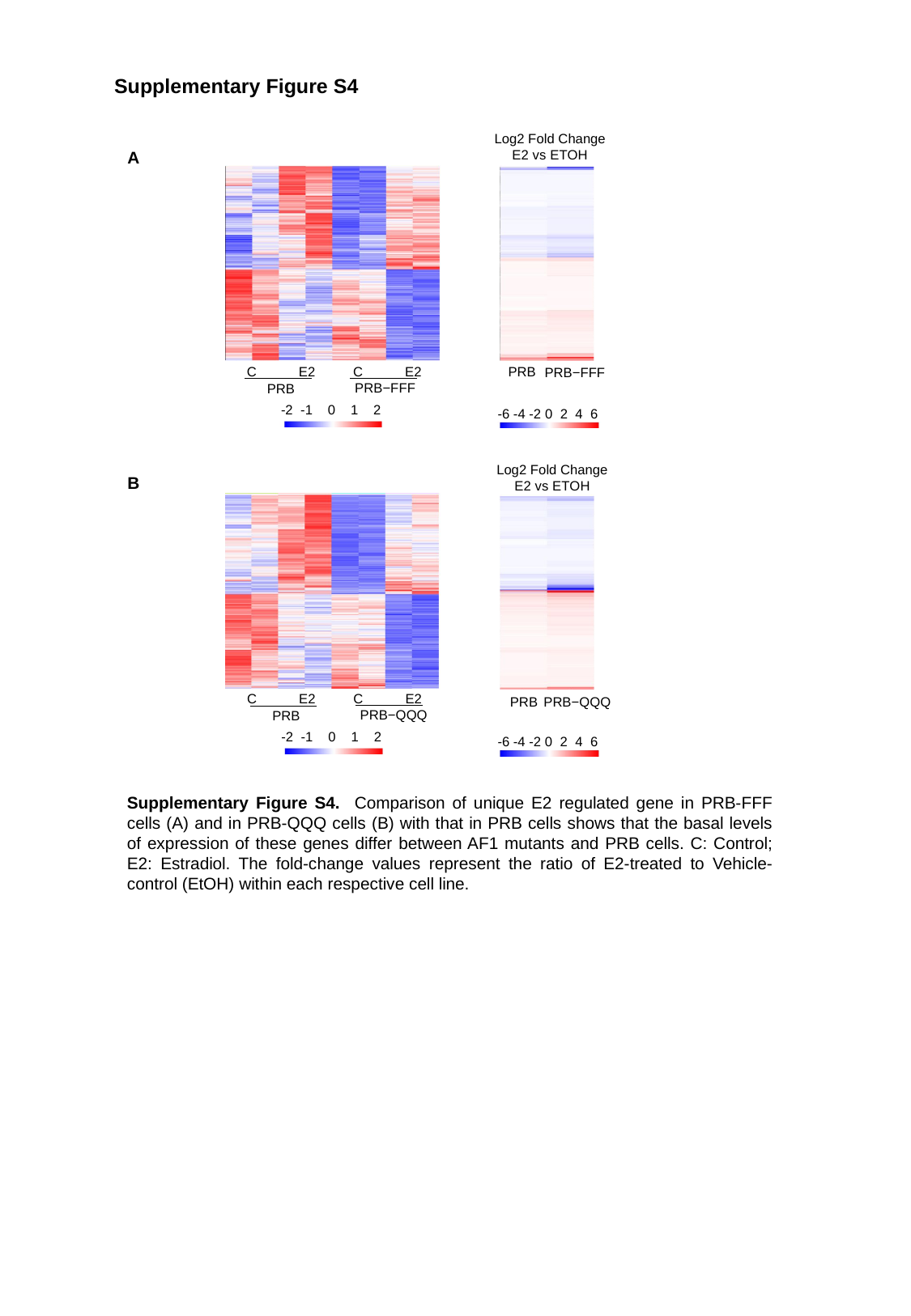

Supplementary Figure S4
Log2 Fold Change
E2 vs ETOH
PRB
PRB−FFF
-6 -4 -2 0 2 4 6
C E2
PRB
C E2
PRB−FFF
-2 -1 0 1 2
A
Log2 Fold Change
E2 vs ETOH
PRB
PRB−QQQ
-6 -4 -2 0 2 4 6
C E2
PRB
C E2
PRB−QQQ
-2 -1 0 1 2
B
Supplementary Figure S4. Comparison of unique E2 regulated gene in PRB-FFF cells (A) and in PRB-QQQ cells (B) with that in PRB cells shows that the basal levels of expression of these genes differ between AF1 mutants and PRB cells. C: Control; E2: Estradiol. The fold-change values represent the ratio of E2-treated to Vehicle-control (EtOH) within each respective cell line.

## Slide 5
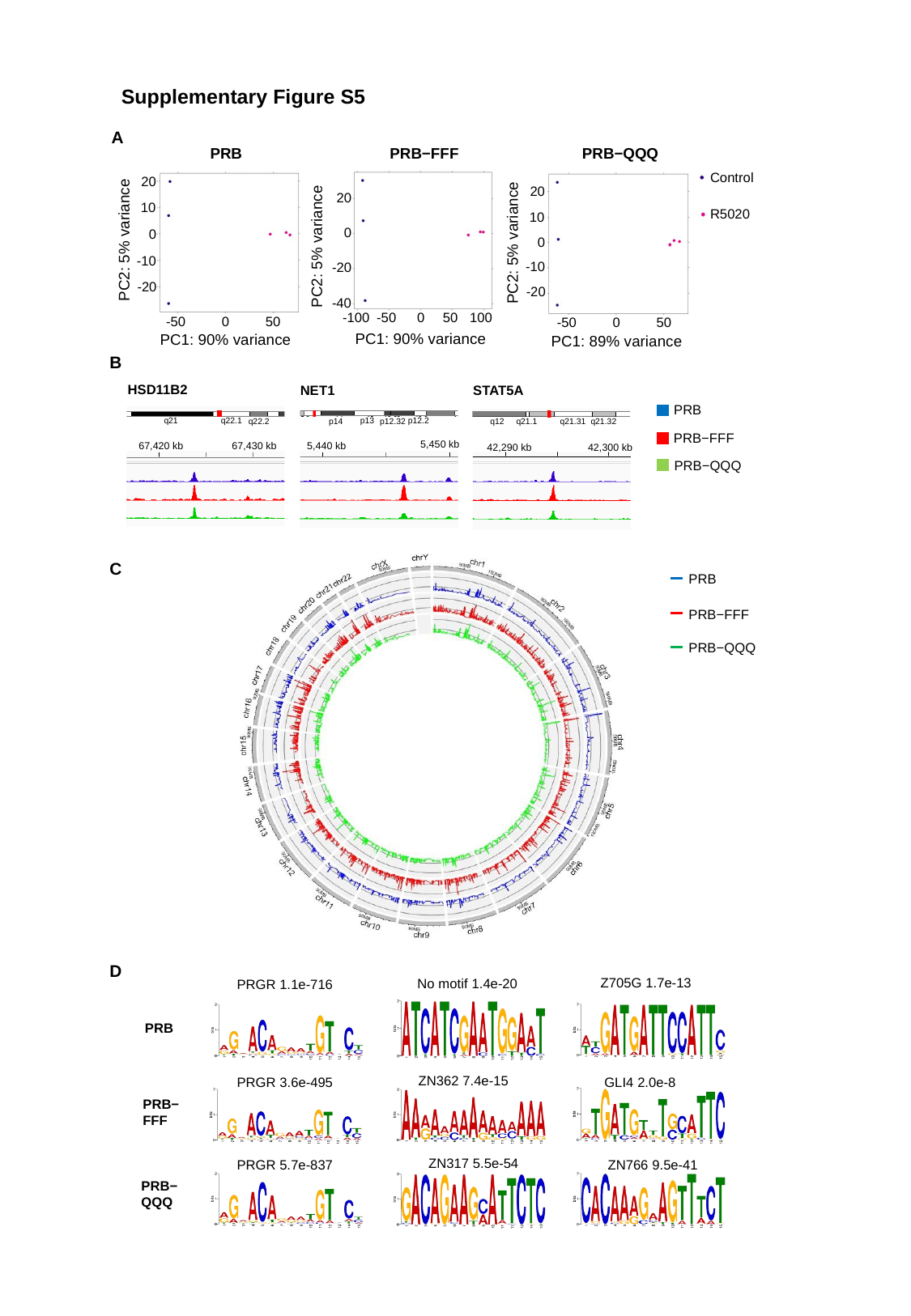

Supplementary Figure S5
A
PRB
PRB−FFF
PRB−QQQ
Control
R5020
20
10
0
PC2: 5% variance
-10
-20
-50
0
50
PC1: 90% variance
20
0
PC2: 5% variance
-20
-40
-100
-50
0
50
100
PC1: 90% variance
20
10
0
PC2: 5% variance
-10
-20
-50
0
50
PC1: 89% variance
B
HSD11B2
q21
q22.1
q22.2
67,420 kb
67,430 kb
NET1
p13
p12.2
p14
p12.32
5,450 kb
5,440 kb
STAT5A
q21.32
q21.31
q12
q21.1
42,290 kb
42,300 kb
PRB
PRB−FFF
PRB−QQQ
C
PRB
PRB−FFF
PRB−QQQ
D
Z705G 1.7e-13
No motif 1.4e-20
PRGR 1.1e-716
PRB
ZN362 7.4e-15
 GLI4 2.0e-8
PRGR 3.6e-495
PRB−FFF
ZN317 5.5e-54
PRGR 5.7e-837
ZN766 9.5e-41
PRB−QQQ

## Slide 6
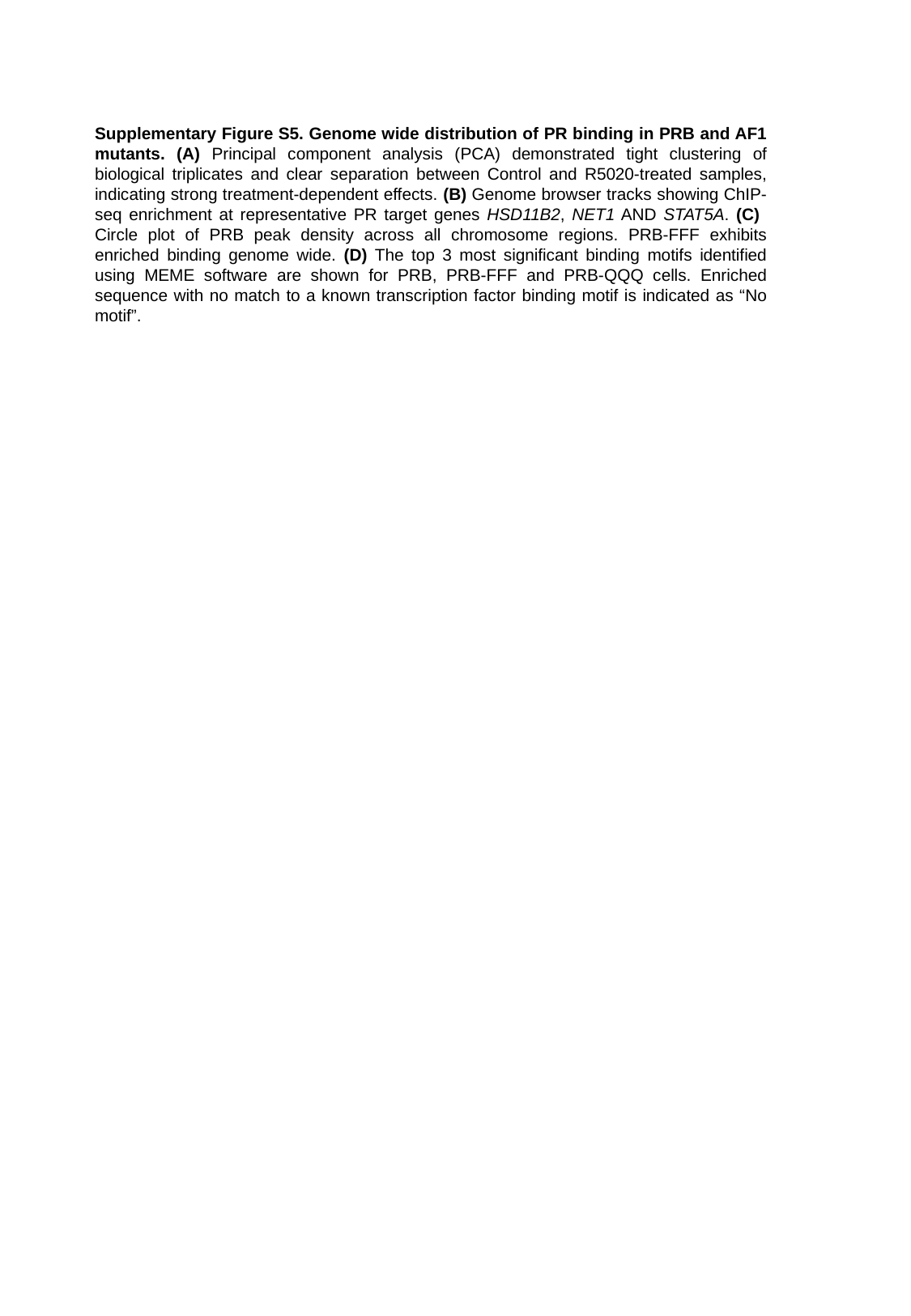

Supplementary Figure S5. Genome wide distribution of PR binding in PRB and AF1 mutants. (A) Principal component analysis (PCA) demonstrated tight clustering of biological triplicates and clear separation between Control and R5020-treated samples, indicating strong treatment-dependent effects. (B) Genome browser tracks showing ChIP-seq enrichment at representative PR target genes HSD11B2, NET1 AND STAT5A. (C) Circle plot of PRB peak density across all chromosome regions. PRB-FFF exhibits enriched binding genome wide. (D) The top 3 most significant binding motifs identified using MEME software are shown for PRB, PRB-FFF and PRB-QQQ cells. Enriched sequence with no match to a known transcription factor binding motif is indicated as “No motif”.

## Slide 7
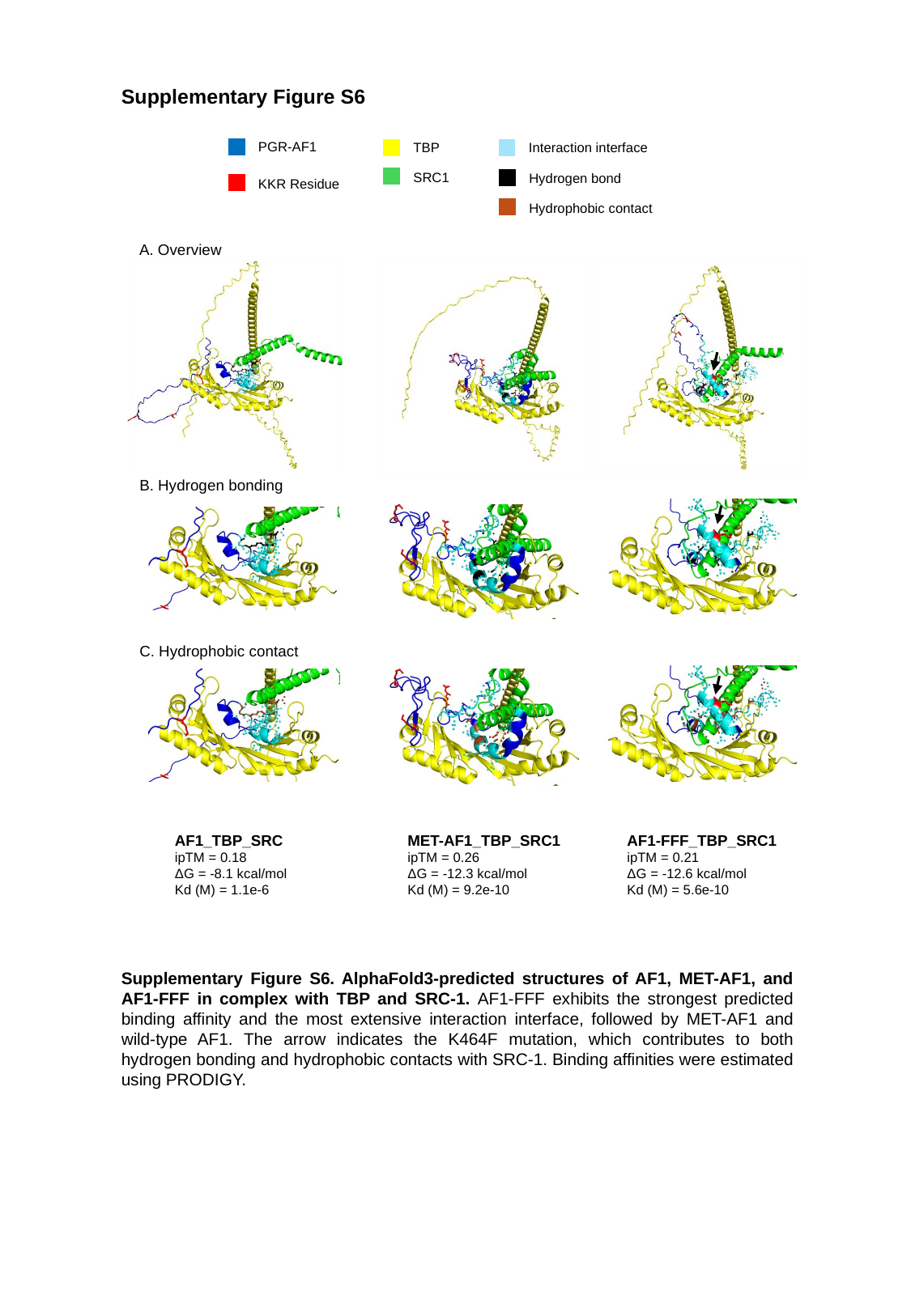

Supplementary Figure S6
PGR-AF1
KKR Residue
TBP
SRC1
Interaction interface
Hydrogen bond
Hydrophobic contact
A. Overview
AF1_TBP_SRC
ipTM = 0.18
ΔG = -8.1 kcal/mol
Kd (M) = 1.1e-6
MET-AF1_TBP_SRC1
ipTM = 0.26
ΔG = -12.3 kcal/mol
Kd (M) = 9.2e-10
AF1-FFF_TBP_SRC1
ipTM = 0.21
ΔG = -12.6 kcal/mol
Kd (M) = 5.6e-10
B. Hydrogen bonding
C. Hydrophobic contact
Supplementary Figure S6. AlphaFold3-predicted structures of AF1, MET-AF1, and AF1-FFF in complex with TBP and SRC-1. AF1-FFF exhibits the strongest predicted binding affinity and the most extensive interaction interface, followed by MET-AF1 and wild-type AF1. The arrow indicates the K464F mutation, which contributes to both hydrogen bonding and hydrophobic contacts with SRC-1. Binding affinities were estimated using PRODIGY.
